# Native T1 mapping in children and young adults with hypertrophic cardiomyopathy

**DOI:** 10.1186/1532-429X-18-S1-Q44

**Published:** 2016-01-27

**Authors:** Keyur Parekh, Michael Markl, Jie Deng, Roger A de Freitas, Cynthia K Rigsby

**Affiliations:** Northwestern University, Chicago, IL USA

## Background

The presence of myocardial fibrosis in hypertrophic cardiomyopathy (HCM) is associated with a wide range of adverse outcomes including ventricular arrhythmia and sudden death in adults. Its presence suggests an adverse prognosis and may provide important therapeutic guidance. Native T1 can be an alternative to routinely used late gadolinium enhancement and has a potential to provide a non-contrast alternative in children.

## Methods

Our cohort population consisted of 21 patients (mean age, 14.1 ± 4.6 years; range 2 - 21 years) with hypertrophic cardiomyopathy undergoing routine clinical cardiac magnetic resonance (CMR). The diagnosis of HCM was based on the demonstration of a non-dilated hypertrophic left ventricle (LV) in the absence of increased LV stress or another cardiac or systemic disease that could result in a similar magnitude of hypertrophy. Twelve subjects (mean age, 15.5 ± 1.6 years; range 2 - 21 years) with low pre-test likelihood of cardiomyopathy, without any known cardiovascular disease, congenital or acquired by history, echocardiography, or CMR served as controls. In all subjects, breath hold, ECG triggered, 2D MOLLI TrueFISP data was acquired in short axis locations at base, mid-chamber, and apex. Native (non-contrast) myocardial T1 maps were calculated a T1 was quantified based in the 16-segment AHA model.

## Results

Global native T1 (averaged over the entire LV) in patients with HCM was significantly increased compared to controls (T1 = 1023.3 ± 41.8 ms vs. 963.5 ± 16.1 ms, p < 0.001). Regional analysis demonstrated significantly elevated (3.8 to 7.6% differences, p < 0.05) native T1 in all 16 AHA segments. A T1 of > 990 msec yielded a sensitivity of 90% and specificity of 90% to identify patients with HCM (p < 0.05). There was a modest but significant correlation between native T1 and indexed LV mass (r = 0.36, p = 0.03). No correlation was identified between native T1 and heart rate, LV ejection fraction, indexed end-diastolic, and end-systolic volume (p = 0.11, 0.82, 0.45, and 0.64). There was no significant difference in T1 values between HCM patients without and with late gadolinium enhancement (p = 0.07).

## Conclusions

Global and segmental native T1 values were elevated in patients with HCM, but did not correlate with parameters of LV structure and function. Native T1 of LV myocardium in children and young adults can be used as a non-invasive tool to identify patients with HCM with a high sensitivity and specificity. Further work should determine whether T1 mapping can guide cardiac risk stratification for children and young adults with HCM.

**Figure 1:**
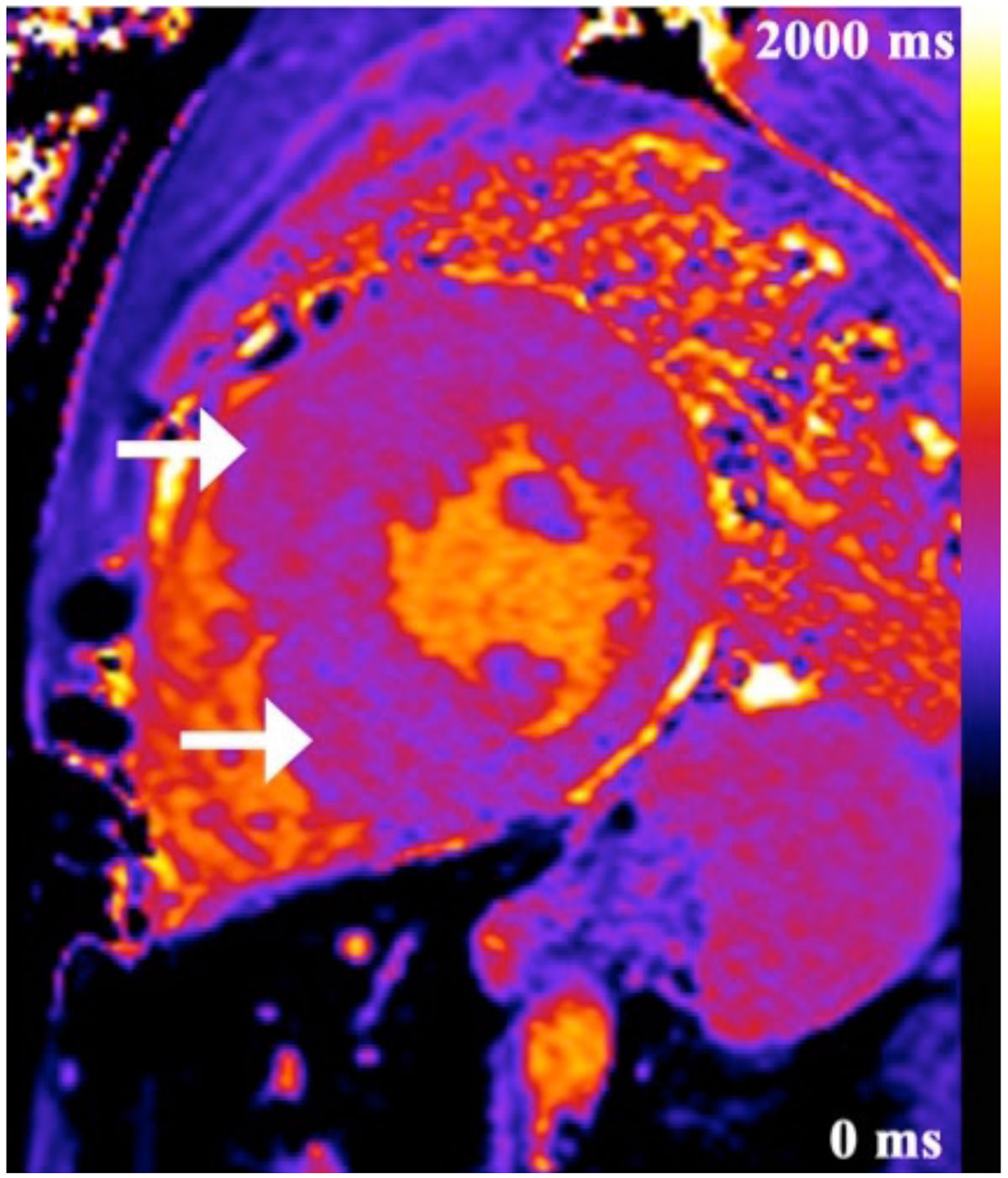
**Example of color-coded T1 map in a patient with hypertrophic cardiomyopathy (HCM)**. Sixteen-year-old male patient presented with palpitations and was diagnosed with HCM on echocardiography. Maximum single wall thickness was 2.6 cm in the mid-chamber antero-septal region. Color-coded T1 map in mid-chamber short axis shows patchy areas of increased T1 relaxation time (arrows), more marked in hypertrophic segments. Color scale ranges from 0 ms to 2000 ms.

